# Patience

**Published:** 1886-11-20

**Authors:** 


### WORDS OF CONSOLATION.

[Communications for this column should be addressed, "Chaplain," care of the Editor of THE HOSPITAL.]

### VIII.—PATIENCE.

HAVING tried to point out the various purposes God has in afflicting different classes of sufferers, we will proceed to consider a little more in detail the special virtues and graces of character which are meant to be perfected, and yet, alas! are sometimes altogether forfeited, through suffering. The first of these is Patience. A sick person does indeed require patience, and patience of many kinds—patience in bearing pain, patience in bearing discomfort, patience in bearing with those around him, with their misunderstandings of his wants and feelings, patience to wait "the appointed time until his change come." Our blessed Lord has said, "In your patience possess ye your souls." (Luke xxi, 19). That is, by patient continuance in well doing, and in enduring tribulation, ye shall keep safe, as the best possession of all, the inner life. That would be safe even were the life of the body lost. Patience is the secret of self-possession; or, rather, he who is the most self-possessed is sure to be the most patient. We speak of people losing temper, losing heart, losing their heads, and so on. What do we mean, but that the better self, the true nature, the power of the kingdom of grace within them has been forfeited for a time? That most precious possession of the Holy Spirit which exercised such sweet influence over the emotions, the affections, and the will has been alienated, and so patience, the guardian angel of the soul's peace, has fled away.

Yet, there must be momentary losses of patience even amongst the most resigned. These are often to be accounted for by their weak physical condition. Great allowances must be made when the nervous system has been completely unstrung, and the once muscular frame debilitated. What wonder if poor souls, usually peaceful and quiet, fail, when through physical infirmity it is impossible to control even restless, fidgety movements of the limbs and body! Oh, ye nurses and attendants, it is part of your high calling to make most ample allowances for the nervous irritability of some of your sufferers. Ye will not judge those to be naturally impatient whose infirmities make even the body and its members so restless and distracted. What wonder if the mind be similarly affected, and tossed to and fro during the storm, like the waves of a troubled sea? Nevertheless, while making every possible allowance for physical infirmity, let us be sure that "patients" best consult their own comfort by cultivating patience. "Tribulation worketh patience" (Rom v, 3). A quiet, calm frame of mind, ever staying itself upon God, is the groundwork of patience. "Why art thou so disquieted, O my soul?" What is the answer? "Put thy trust in God" (Psalm xlii). Whenever you feel your patience threatening to leave you it is a good thing to make a decided act of faith in God. Simply say, "I believe in God." That one short appeal for help will often baffle the enemy and re-establish the soul in full possession. Very sweet is the return of the angel of patience, sweet and refreshing to the sufferer, encouraging to the attendants, and a great example to others in hospital who have yet to learn how to become better "patients."

*(To be continued.)*

